# Regorafenib is suitable for advanced colorectal cancer patients who have previously received trifluridine/tipiracil plus bevacizumab

**DOI:** 10.1038/s41598-023-29706-6

**Published:** 2023-02-10

**Authors:** Toshihiko Matsumoto, Tatsuki Ikoma, Shogo Yamamura, Kou Miura, Takao Tsuduki, Takanori Watanabe, Hiroki Nagai, Masahiro Takatani, Hisateru Yasui

**Affiliations:** 1 Department of Clinical Oncology, Kobe City Medical Center General Hospital, 2-1-1, Minatojima Minamimachi, Chuo-Ku, Kobe, Hyogo 6500047 Japan; 2 Department of Internal Medicine, Himeji Red Cross Hospital, 1-12-1, Shimoteno, Himeji, Hyogo 6708540 Japan; 3 Department of Surgery, Himeji Red Cross Hospital, 1-12-1, Shimoteno, Himeji, Hyogo 6708540 Japan; 4 Cancer Treatment Center, Kansai Medical University, 2-3-1, Hirakatashinmachi, Hirakata, Osaka 573-1191 Japan

**Keywords:** Cancer therapy, Gastrointestinal cancer, Metastasis

## Abstract

Regorafenib is a standard salvage line therapy used for advanced colorectal cancer (CRC). Recently, trifluridine/tipiracil (TFTD) plus bevacizumab also showed promising efficacy as a salvage line therapy for advanced CRC. However, the efficacy and safety of regorafenib for patients with advanced CRC who have previously received TFTD plus bevacizumab is unclear. We retrospectively collected clinicopathologic data from patients with advanced CRC who received regorafenib after TFTD plus bevacizumab in multiple institutions between April 2017 and June 2020.Thirty-four advanced CRC patients who received regorafenib were analyzed. The median age was 66.5 (range 43–81 years), 11 patients were male, and all had an ECOG performance status(PS) of 0 or 1. Twenty-two patients had left-sided tumors, 18 patients had RAS mutants, and 1 patient had a BRAF V600E mutation. The response rate was 0%, and the disease control rate was 31%. The median progression-free survival was 70 days (95% CI: 56–91), and the overall survival was 233 days (95% CI: 188–324). Treatment was discontinued in 32 patients, and 28 (82%) discontinued treatment due to progressive disease. The major grade 3 and4 toxicities were proteinurea (29%), hypertension (26%), hand-foot syndrome(15%), and platelet decrease (6%). Regorafenib after TFTD plus bevacizumab showed efficacy similar to that of the previous study, and no new adverse events were observed.

## Introduction

Colorectal cancer (CRC) is the third leading cause of cancer-related deaths^1^. Cytotoxic chemotherapy should be combined with EGFR-targeted antibodies in patients with RAS wild-type metastatic CRC (mCRC). In patients with RAS-mutated mCRC, cytotoxic chemotherapy combined with bevacizumab, ramucirumab, or aflibercept is recommended as a first-line or second-line therapy. In patients with the BRAF V600E mutant mCRC, encorafenib plus cetuximab is recommended as a second-line therapy. After these therapies, regorafenib and trifluridine/tipiracil (TFTD) are the standard regimens.

Regorafenib is the only multikinase agent approved for mCRC patients with disease progression after the other standard treatments. Regorafenib showed significant improvement of overall survival (OS) in the CORRECT and CONCUR trials^2,3^. TFTD is an oral combination of the thymidine-based nucleoside analogs trifluridine and tipiracil hydrochloride at a molar ratio of 1:0.5. TFTD also showed significant improvement of OS in the RECOURSE and TERRA trials^4,5^. Both drugs are used as standard chemotherapy treatments for patients with mCRC who had disease progression after the other standard treatments.

Recently, TFTD plus bevacizumab showed promising progression-free survival (PFS) and OS in several phase 2 trials. Kuboki et al. reported the CTASK-FORCE trial, in which with TFTD plus bevacizumab (Bmab), the median progression-free survival (PFS) was 5∙6 months (95% confidence interval(CI): 3·4–7·6) and the median overall survival (OS) was 11·4 months (95% CI 76–139)^6^. Pfeiffer et al. reported a randomized phase 2 trial of TFTD plus Bmab compared with TFTD monotherapy. TFTD plus Bmab showed significant improvement in PFS (4.6 vs. 2.6 months, hazard ratio [HR]: 0.45; 95% CI 0.29–0.72) in mCRC patients receiving refractory standard therapy^7^. Other single-arm phase 2 trials showed similar efficacy to TFTD plus Bmab^8^. Thus, TFTD + Bmab is now one of the standard regimens used for mCRC as a late line therapy.

Efficacy data of regorafenib in mCRC patients previously treated with TFTD + Bmab are lacking. We thus retrospectively evaluated the efficacy and safety of regorafenib in such patients.

## Methods

### Patients

This was a multicenter retrospective study conducted at two institutions (Himeji Red Cross Hospital, Himeji, Hyogo, Japan; Kobe City Medical Center General Hospital, Kobe, Hyogo, Japan). The analysis of this study was based on our previous study^9^. We retrospectively collected the clinical data of patients with mCRC treated with regorafenib between April 2017 and June 2020. All data were collected retrospectively from electronic medical records. All procedures were performed in accordance with institutional and national standards on human experimentation, as confirmed by the ethics committee of Himeji Red Cross Hospital and Kobe City Medical Center General Hospital, in accordance with the Declaration of Helsinki of 1964 and its later amendments.

The inclusion criteria were as follows: (1) unresectable colorectal cancer, (2) histologically proven colorectal carcinoma, (3) refractory or intolerant to TFTD plus bmab and (4) no prior administration of regorafenib. The study protocol was approved by the Institutional Review Board of Himeji Red Cross Hospital and Kobe City Medical Center General Hospital.

### Treatment

The patients received regorafenib doses of 160 mg, 120 mg, and 80 mg that were administered orally once daily for the first 3 weeks of each 4 week cycle until disease progression, unacceptable adverse events and death.

### Evaluation and statistical analysis

The ECOG performance status was defined by medical oncologists and chemotherapeutic nurses. Tumor response was evaluated according to the Response Evaluation Criteria in Solid Tumors (RECIST) version 1.1. Toxicity was assessed using the Common Terminology Criteria for Adverse Events (CTCAE) version 4.1. PFS (progression free survival) was defined as the time from the date of regorafenib initiation to the date of disease progression or death from any cause. Patients for whom there was no information regarding tumor progression were treated as censored cases. OS (overall survival) was defined as the time from the date of regorafenib initiation to the date of death from any cause. Patients for whom there was no information regarding tumor progression were treated as censored cases. OS and PFS were estimated using the Kaplan–Meier method. Statistical analyses were performed using JMP version 12 (SAS Institute Inc., Cary, NC, USA).

### Ethics approval and consent to participate

This study was approved by the Institutional Review Board of Kobe City Medical Center General Hospital and Himeji Red Cross Hospital. All procedures performed in studies involving human participants were in accordance with the ethical standards of the institutional review board of the Kobe City Medical Center General Hospital and Himeji Red Cross Hospital and with the 1964 Helsinki declaration and its later amendments or comparable ethical standards. Given that this was an observational study, the Institutional Review Board of Kobe City Medical Center General Hospital and Himeji Red Cross Hospital waived the need of informed consent for this study. However, we guaranteed the opportunity of opt-out. Obtaining consent in this way was approved by the ethics committee of Kobe City Medical Center General Hospital and Himeji Red Cross Hospital. Our team acquired administrative permission to access the data used in this research.

## Results

### Baseline characteristics

Clinical data were collected from 34 patients with CRC who had been treated with regorafenib. Their characteristics are presented in Table 1. The median patient age was 66 years (range: 43–81 years), and eight patients (24%) had an ECOG PS of 0. Eighteen patients (53%) had RAS mutations, one patient (3%) had a BRAF V600E mutation, and 23 patients (68%) had two or more metastatic sites. Twenty-nine patients (85%) received two or more prior chemotherapy regimens, and 28 patients (78%) received regorafenib immediately after the TFTD + Bmab refractory treatment.

**Table 1:** Patients characteristics.

Age	Median(range)	66.5 (43–81)
Sex	Male	11 (32%)
ECOG PS	0/1	8 (24%)/26 (76%)
Tumor location	Right/left	11 (32%)/23 (68%)
RAS status	Mutant	18 (53%)
BRAF status	V600E mutant	1 (3%)
MSI status	MSS	24 (71%)
	Unknown	10 (29%)
Resection of primary tumor	Yes	27(79%)
Number of metastatic organs	≧2	23 (68%)
Liver metastasis	Yes	17 (50%)
Lung metastasis	Yes	23 (68%)
Peritoneal dissemination	Yes	11 (32%)
Starting dose	120 mg	24 (71%)
	160 mg	5 (15%)
	80 mg	5 (15%)
Number of prior chemotherapy	2	5 (15%)
	3	11 (32%)
	≧ 4	18 (53%)
Prior treatment	5-FU	34 (100%)
	Oxaliplatin	33 (97%)
	CPT-11	31 (91%)
	Anti VEGF drug	34 (100%)
	Anti EGFR antibody	16 (47%)

*ECOG* Eastern Cooperative Oncology Group, *PS* performance status, *MSI* microsatellite instability, *MSS* microsatellite stable5-FU, 5-fluorouracil, *CPT-11* irinotecan, *VEGF* vascular endothelial growth factor, *EGFR* epidermal growth factor receptor.

The starting dose of regorafenib for 24 of the patients (71%) was 120 mg, for 5 patients (15%) it was 160 mg and 80 mg, respectively. In the patients receiving 120 mg and 160 mg doses, 19 patients (79%) and 4 patients (80%) required dose reductions. For the patients receiving the 80 mg doses, one patient (20%) required a dose reduction and one patient (20%) required a dose increase.

### Efficacy

**Figure 1:**
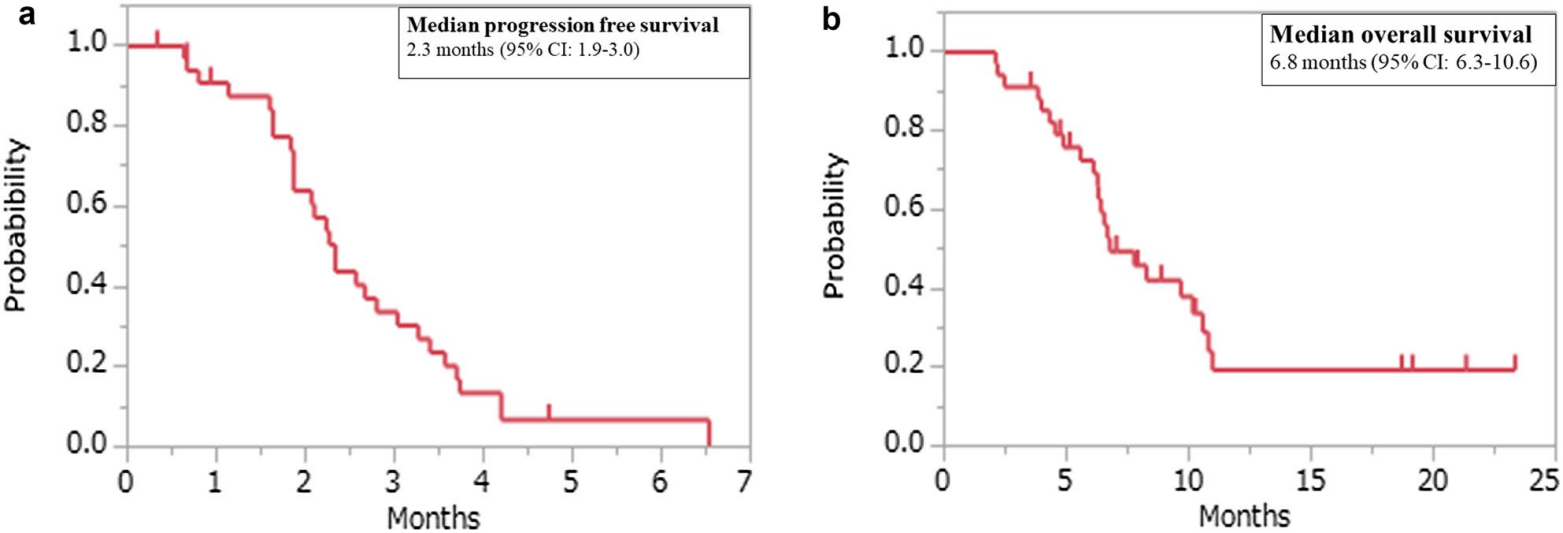
Kaplan–Meier plots of (**a**) progression-free survival (PFS) and (**b**) overall survival (OS) among the study participants.

Of the 29 (85%) patients with measurable lesions, no patients achieved a complete response or partial response, 9 patients showed stable disease, resulting in a response rate (RR) of 0% and a disease control rate (DCR) of 28%. After a median follow-up period of 6.6 months, the median PFS was 2.3 months (95% CI 1.9–3.0) and the median OS was 6.7 months (95% CI 6.3–10.6) (Fig. 1).

**Figure 2:**
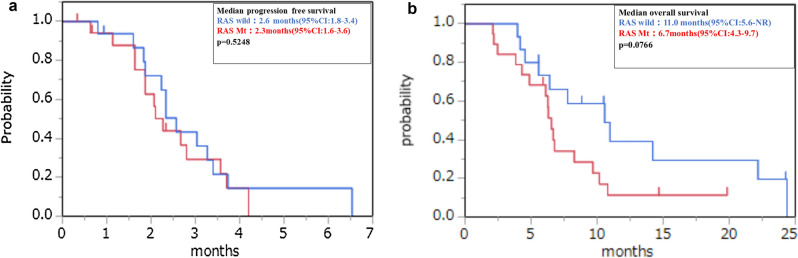
Kaplan–Meier plots of (**a**) progression-free survival (PFS) and (**b**) overall survival (OS) among study participants. Red line: RAS wild group, Blue line: RAS mutant group.

**Figure 3:**
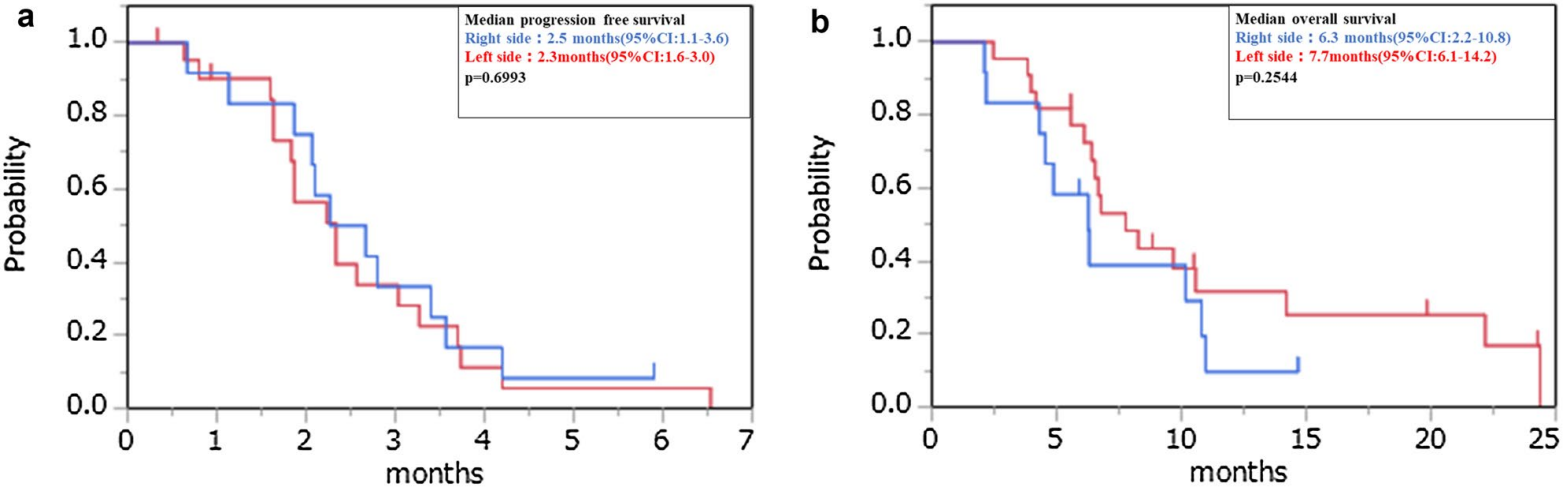
Kaplan–Meier plots of (**a**) progression-free survival (PFS) and (**b**) overall survival (OS) among study participants. Red line: Left side tumor; Blue line: Right side tumor.

**Figure 4:**
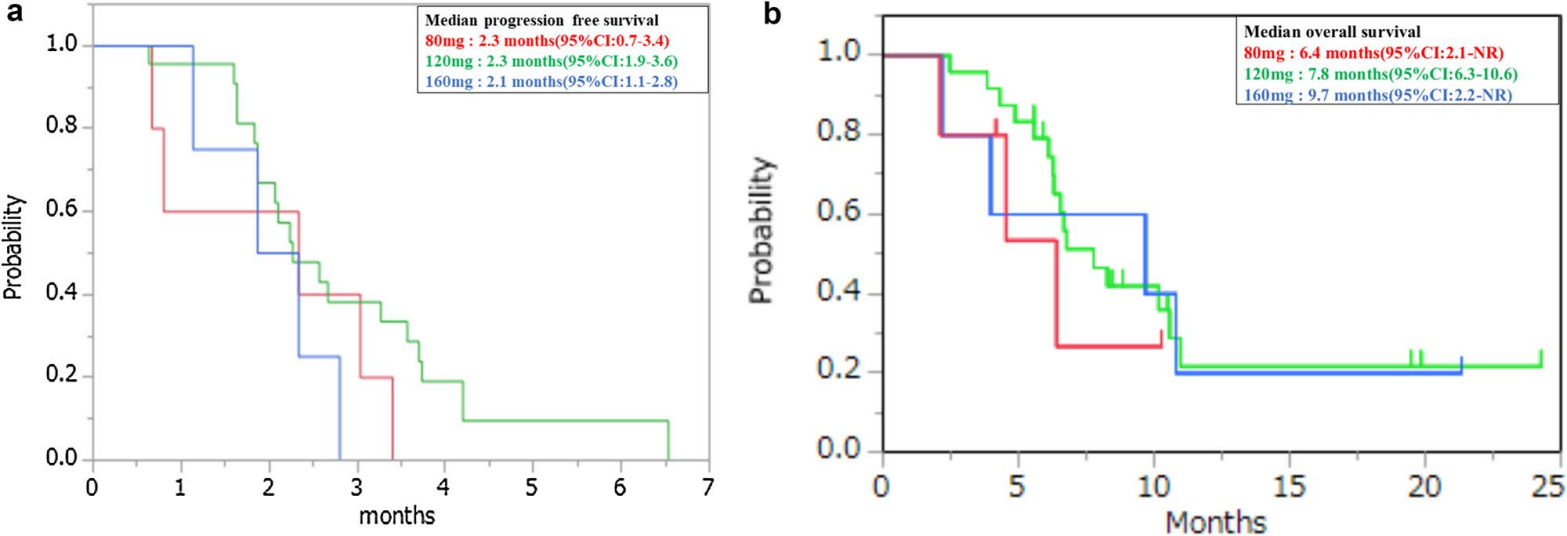
Kaplan–Meier plots of (**a**) progression-free survival (PFS) and (**b**) overall survival (OS) among study participants. Red line: 160 mg group; Green line: 120 mg group; Blue line: 80 mg group.

In RAS wild patients, the median PFS was 2.6 months (95% CI 1.8–3.4) and the median OS was 11.0 months (95% CI 5.6-not reached). In RAS mutant patients, the median PFS was 2.3 months (95% CI 1.6–3.6) and the median OS was 6.7 months (95% CI 4.3–9.7). There was no significant difference according to the RAS status (Fig. 2). We also examined the correlation between primary tumor location and efficacy, but no difference was found in median PFS and median OS when comparing the left and right sides (Fig. 3).We examined the effect of the starting dose, but there were no significant differences between the 80, 120, and 160 mg treatments (Fig. 4).

### Safety

The adverse events among the study participants are shown in Table 2. The major severe adverse events (≥ grade 3) were hypertension (26%), hand foot syndrome (15%), proteinuria (6%), platelet count (6%), colitis (6%), and liver dysfunction (3%). There was no significant difference in safety between the starting doses. Two patients were on ongoing treatment, and 32 patients discontinued treatment, of which 28 (88%) were due to disease progression and 4 (13%) due to adverse events. No treatment-related deaths were observed and no new adverse events were observed.

**Table 2:** Adverse events.

	All	≦ Grade 2	≧ Grade 3	
(a) All patients				
Hypertension	12 (35%)	3 (9%)	9 (26%)	
Hand foot syndrome	25 (75%)	20 (59%)	5 (15%)	
Proteinuria	13 (38%)	11 (32%)	2 (6%)	
Platelet decreased	8 (24%)	6 (18%)	2 (6%)	
Colitis	4 (12%)	2 (6%)	2 (6%)	
Liver dysfunction	6 (18%)	5 (15%)	1 (3%)	
Fatigue	12 (35%)	12 (35%)	0	
Hypotyroidism	1 (3%)	1 (3%)	0	
Hoarseness	14 (41%)	14 (41%)	0	
Stomatitis	3 (9%)	3 (9%)	0	

Starting dose	All	80 mg (n = 5)	120 mg (n = 24)	160 mg (n = 5)
(b) Starting dose subgroup analysis				
Proteinuria	2 (6%)	1 (20%)	1 (4%)	0
Hypertension	9 (26%)	0	6 (25%)	3 (60%)
Hand foot syndrome	5 (15%)	1 (20%)	3 (13%)	1 (20%)
Platelet decreased	2 (6%)	0	1 (4%)	1 (20%)
Colitis	2 (6%)	0	2 (8%)	0
Liver dysfunction	1 (3%)	0	1 (4%)	0

## Discussion

As a salvage line chemotherapy for mCRC, regorafenib showed a median PFS of 1.9 months and a median OS of 6.4 months in the CORRECT trial. In the Asian population, the CONCUR trial showed that it had a median PFS of 3.2 months and a median OS of 8.8 months. Our study showed similar efficacy (median PFS of 2.3 months and median OS of 6.7 months) despite the failure of the TFTD plus Bmab therapy (Table 3)^10,11^. The disease control rate in this study was 31%. The disease control rate in the placebo arm was 18% in the CORRECT trial and 7% in the CONCUR trial. The subject of our study is the patients after receiving TFTD + Bmab, and the prognosis is judged to be worse than that of the subject of both studies. We therefore determined that regorafenib has a modest efficacy for those population. In RAS wild patients, the median PFS was 2.6 months, and the median OS was 11 months. In RAS mutant patients, the median PFS was 2.3 months, and the median OS was 6.7 months. There was no statistically significant difference between the RAS wild and RAS mutant patients. These results suggest that regorafenib is a therapeutic option for mCRC patients who previously received TFTD plus Bmab, despite their RAS status. Nakajima et al. reported that primary rumor location is not a prognostic and predictive factor in patients with mCRC who received regorafenib or TFTD therapy^12^. In our study, regorafenib showed similar efficacy regarding primary tumor location. These results suggested that regorafenib may be effective as late line chemotherapy for mCRC regardless of primary tumor location.

**Table 3:** Efficacy of regorafenib as a salvage line chemotherapy for colorectal cancer.

Study name	This study	REGOTAS^10^	Ogata et al.^11^	CORRECT^2^	CONCUR^3^
Study type	Retrospective	Retrospective	Retrospective	Phase 3	Phase 3
N	34	223	57	505	136
Age	66.5 (43–81)	64 (31–84)	66 (41–81)	61 (54–67)	57.5 (50–66)
Prior regimens≧3	85%	48%	56%	74%	62%
Prior TFTD	100%	0%	32%	0%	0%
Response rate	0%	0%	2%	1%	4%
Disease control rate	31%	32%	32%	41%	51%
Progression free survival (months)	2.3	2.1	2	1.9	3.2
Overall survival (months)	6.7	7.9	9.9	6.4	8.8

*TFTD* trifluridine/tipiracil.

In our study, the median OS after the 1st line chemotherapy was 40.1 months (95% CI 29.8–124.7). These results tended to be better than those in recent Phase 3 trials for chemotherapy-naïve mCRC patients^13–17^. Moreover, the median OS after the first administration of TFTD plus Bmab was 12.8 months (95% CI 12.3–15.7). Ogata et al. reported a multi-institutional retrospective study which found that the sequential use of TFTD and regorafenib may prolong survival in mCRC patients^10^. Grothey et al. reported the strategy of administering 5-FU, oxaliplatin, and irinotecan to all patients with mCRC who were candidates for such therapy^18^. Our study suggests that the sequential use of TFTD plus Bmab and regorafenib may prolong survival in patients with mCRC.

In our study, 76% of all patients had ECOG PS 1, which was a worse population than the CORRECT trial (PS 1 was 48%). However, the profile of adverse events was similar between the CORRECT trial and our study. In our study, 53% of patients received four or more chemotherapy regimens before regorafenib, and the most common severe (≥ Grade3) adverse events were hypertension (26%) and hand foot syndrome (15%). This suggests that regorafenib is tolerant of mCRC refractory to heavy chemotherapy regimens containing TFTD + Bmab.

The standard dose of regorafenib monotherapy was 160 mg daily for the first 3 weeks of each 4-week cycle in the CORRECT and CONCUR trials. However, in the CORRECT and CONCUR trials, 76% and 71% of the patients required dose modifications. Bekaii-Sabb et al. reported a randomized phase 2 study of the dose-escalation dosing strategy, which represents an alternative approach for mCRC patients as salvage line setting^19^. In our study, 15% of patients received 160 mg as the starting dose, 71% received 120 mg, and 15% received 80 mg. No clear correlation was found between the starting dose and the effect. The groups with starting doses of 120 mg and 160 mg tended to have more serious adverse events than those receiving 80 mg. No patients in the 120 and 80 mg groups were able to increase their doses after the start of the treatment.

This study focused on the efficacy and safety of regorafenib in patients with mCRC who previously received TFTD plus Bmab. To the best of our knowledge, this is the first study on regorafenib for such patients. The phase III TRUSTY trial is currently underway to confirm the non-inferiority of TFTD plus Bmab to S-1 plus irinotecan/FOLFIRI plus Bmab in patients with unresectable refractory colorectal cancer and those who are intolerant to first-line fluoropyrimidines, OX, Bmab, and anti-EGFR antibodies. Furthermore, the randomized phase II TASCO 1 trial was conducted to evaluate the efficacy of TFTD plus Bmab when compared with capecitabine plus Bmab in patient’s intolerant to IRI- or OX-based chemotherapy and those who were unlikely to be cured according to the investigators’ judgement; this showed a favorable primary outcome for PFS of 7.82 months vs 9.23 months (HR = 0.71, 95% CI 0.48–1.06)^20^. A phase III SOLISTICE trial to evaluate TFTD plus Bmab when compared to capecitabine plus Bmab as a first-line therapy in elderly patients with unresectable colorectal cancer is currently underway. Recently, in a phase III SOLISTICE trial, TFTD plus Bmab showed almost similar PFS to capecitabine plus Bmab as a first-line therapy in elderly patients with unresectable colorectal cancer (9.4 months vs. 9.3 months, Hazard ratio: 0.87. 95% confidence interval: 0·75–1·02; *p* = 0·0464)^21^. A phase III study of TFTD in combination with bevacizumab vs TFTD single agent in patients with refractory metastatic colorectal cancer (SUNLIGHT) is ongoing (NCT04737187). It is important that we explore the efficacy of regorafenib after TFTD + Bmab treatments in mCRC patients.

This study had several limitations. As it was a retrospective study. On the other hand, our study is the only one study to investigate efficacy of regorafenib after administration of TFTD plus Bmab. Our efficacy and safety dates were comparable to those of the regorafenib arm of the CORRECT and CONCUR trials. The results indicate that regorafenib has a similar efficacy and safety in refractory or intolerant TFTD plus Bmab patients with mCRC when compared with previous studies.

## Conclusions

In conclusion, regorafenib after TFTD plus Bmab showed an efficacy similar to that in a previous study, and no new adverse events were observed. Sequential use of TFTD plus Bmab and regorafenib may prolong survival in patients with mCRC. Further prospective trials are required.

## Data Availability

All the data and materials supporting the conclusions are included in the main paper. The datasets used in the current study are available from the corresponding author upon request.

## Abbreviations

mCRC: Metastatic colorectal cancer
TFTD: Trifluridine/tipiracil
Bmab: Bevacizmab
CTCAE: Common terminology criteria for adverse events
DCR: Disease control rate
ECOG: Eastern cooperative oncology group
CI: Confidence interval
OS: Overall survival
PFS: Progression-free survival
PS: Performance status
RECIST: Response evaluation criteria in solid tumors
RR: Response rate
EGFR: Epidermal growth factor receptor
VEGF: Vascular endothelial growth factor
