# Author Correction: Regorafenib is suitable for advanced colorectal cancer patients who have previously received trifluridine/tipiracil plus bevacizumab

**DOI:** 10.1038/s41598-023-39009-5

**Published:** 2023-07-24

**Authors:** Toshihiko Matsumoto, Tatsuki Ikoma, Shogo Yamamura, Kou Miura, Takao Tsuduki, Takanori Watanabe, Hiroki Nagai, Masahiro Takatani, Hisateru Yasui

**Affiliations:** 1 Department of Clinical Oncology, Kobe City Medical Center General Hospital, 2‑1‑1, Minatojima Minamimachi, Chuo‑Ku, Kobe, Hyogo 6500047 Japan; 2 Department of Internal Medicine, Himeji Red Cross Hospital, 1‑12‑1, Shimoteno, Himeji, Hyogo 6708540 Japan; 3 Department of Surgery, Himeji Red Cross Hospital, 1‑12‑1, Shimoteno, Himeji, Hyogo 6708540 Japan; 4 Cancer Treatment Center, Kansai Medical University, 2‑3‑1, Hirakatashinmachi, Hirakata, Osaka 573‑1191 Japan

Correction to: *Scientific Reports* https://doi.org/10.1038/s41598-023-29706-6, published online 10 February 2023

The original version of this Article contained errors.

In Figure 2, panel (**b**), the scale of the y-axis was misaligned.

In addition, in Figure 4, panel (**b**) was a duplication of panel (**a**).

The original Figures 2 and 4 and accompanying legends appear below.

**Figure 2:**
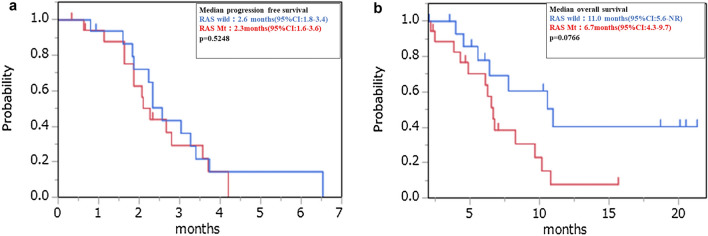
Kaplan–Meier plots of (**a**) progression-free survival (PFS) and (**b**) overall survival (OS) among study participants. Red line: RAS wild group, Blue line: RAS mutant group.

**Figure 4:**
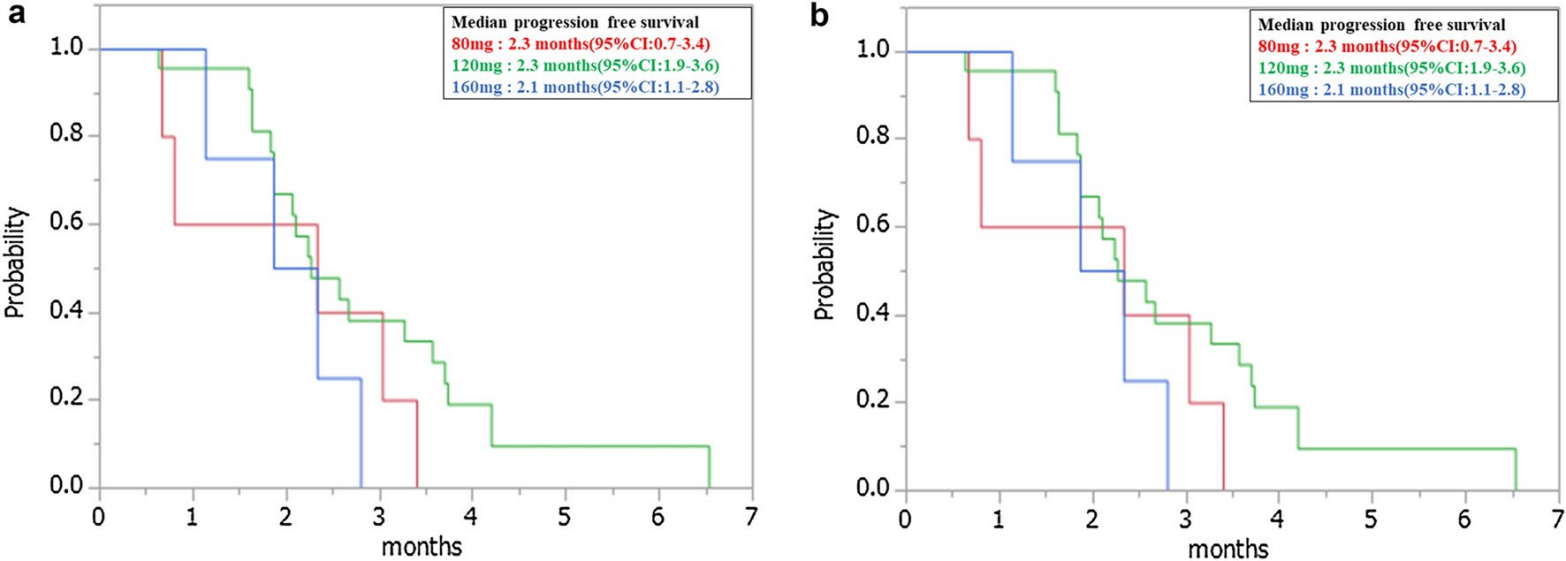
Kaplan–Meier plots of (**a**) progression-free survival (PFS) and (**b**) overall survival (OS) among study participants. Red line: 160 mg group; Green line: 120 mg group; Blue line: 80 mg group.

The original Article has been corrected.

